# Mutation in the loop C-terminal to the cyclophilin A binding site of HIV-1 capsid protein disrupts proper virus assembly and infectivity

**DOI:** 10.1186/1742-4690-4-19

**Published:** 2007-03-19

**Authors:** Samir Abdurahman, Stefan Höglund, Anders Höglund, Anders Vahlne

**Affiliations:** 1 Division of Clinical Microbiology, Karolinska Institutet, Karolinska University Hospial, Stockholm, Sweden; 2 Department of Biochemistry, Biomedical Center, Uppsala University, Uppsala, Sweden

## Abstract

We have studied the effects associated with two single amino acid substitution mutations in HIV-1 capsid (CA), the E98A and E187G. Both amino acids are well conserved among all major HIV-1 subtypes. HIV-1 infectivity is critically dependent on proper CA cone formation and mutations in CA are lethal when they inhibit CA assembly by destabilizing the intra and/or inter molecular CA contacts, which ultimately abrogate viral replication. Glu98, which is located on a surface of a flexible cyclophilin A binding loop is not involved in any intra-molecular contacts with other CA residues. In contrast, Glu187 has extensive intra-molecular contacts with eight other CA residues. Additionally, Glu187 has been shown to form a salt-bridge with Arg18 of another *N*-terminal CA monomer in a *N-C *dimer. However, despite proper virus release, glycoprotein incorporation and Gag processing, electron microscopy analysis revealed that, in contrast to the E187G mutant, only the E98A particles had aberrant core morphology that resulted in loss of infectivity.

## Findings

The HIV-1 capsid protein (CA, p24) is the building block of the conical core structure of the virus. It is initially produced as a part of the Gag precursor (p55) and during or concomitant with the virus release, p55 is cleaved sequentially into the matrix (MA; p17), capsid, nucleocapsid (NC; p7) and p6 proteins [1,2]. Capsid protein consists of two independently folded globular domains, the *N*-and *C*-terminal domain [3] connected through a short flexible hinge region.

Several studies have shown that mutations within the *gag *gene disrupt virus replication or infectivity [4-8] and the infectivity of HIV-1 is critically dependent on proper CA assembly and disassembly following cell entry [9]. Although much of the assembly properties of HIV-1 CA were based on x-ray crystallographic data, NMR and *in vitro *assembly models, the importance of major homology region [10], the binding site for cyclophilin A (CypA) [11,12], and the CA dimer interfaces [13,14] are some of the functions in CA that have been characterised using mutational analysis.

**Figure 1:**
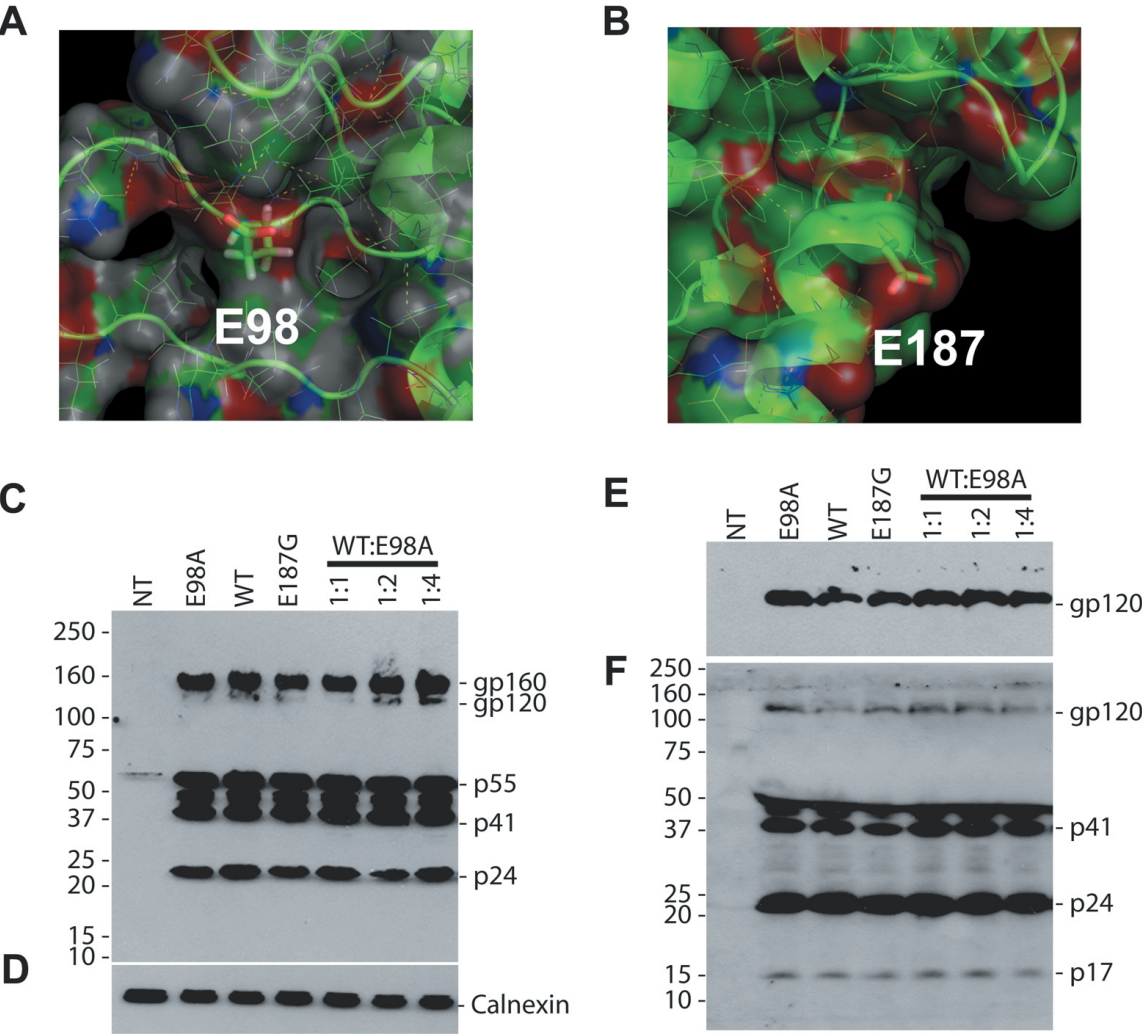
Structural view and Western blot analysis of capsid mutants. A close view of the structure of the cyclophilin A binding loop in the *N*-terminal (A) and the position of E187 in the *C*-terminal (B) HIV-1CA domains. The two residues in this study, E98 and E187, are being explicitly highlighted. The figure was produced with PyMOL [27] and the structure was obtained from the Protein Data Bank (cf PDB entry 1E6J [3]). (C to F) Western blot analysis of mutant and wild-type pNL4-3 transfected cells (C and D), and viral lysates (E and F). HeLa-tat cells were transfected as indicated with 2 μg of proviral DNAs using the non-liposomal FuGENE transfection reagent (Roche) as recommended by the manufacturer. Cells were also co-transfected with mutant and wild-type pNL4-3 as indicated. Forty-eight to 72 hrs post-transfection, cells were harvested and proteins were separated by SDS-PAGE in 4–12% gels and transferred to a nitrocellulose membrane. The membranes were initially probed with HIV+ patient serum (C and F) and were then reprobed with rabbit anti-calnexin antibody (D) or mouse monoclonal anti-V3 antibody (E) using horseradish peroxidase-conjugated secondary antibodies raised against mouse (DAKO, 1:4000), human (Pierce, 1:20,000), or rabbit (Sigma, 1:4,000) IgG. The protein bands were visualized by chemiluminescence. The positions of specific viral proteins are indicated to the right. Numbers to the left depict positions of molecular mass markers in kDa.

Most of amino acid sequences in the CypA-binding loop of HIV-1 CA have been investigated using both genetic and structural studies [12,15-17]. However, Glu98 which is well conserved [18] among all major HIV-1 subtypes was not previously investigated. Glu98 is located on a surface C-terminal to the CypA-binding site and has no intra-molecular contacts with other residues except for a single hydrogen bond with Arg100 [19]. In sharp contrast, Glu187 has extensive contacts with eight other CA residues (Fig 1A and 1B).

**Figure 2:**
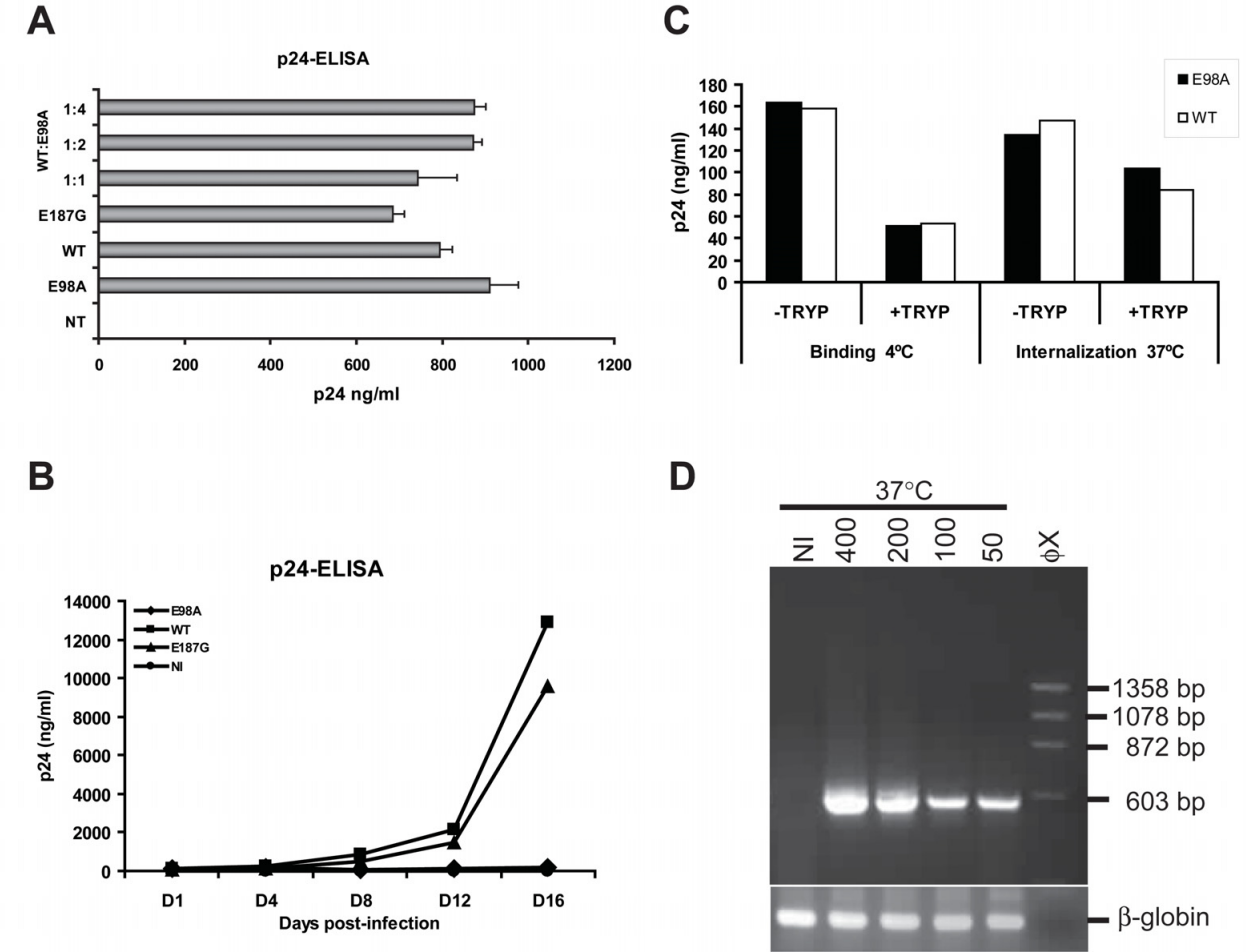
Virus release and internalization studies. p24-ELISA of transfected 293T cell (A) and infected H9 cell (B) culture supernatants. (A) 293T cells were transfected or co-transfected with mutant and wild-type pNL4-3 (2 μg) as indicated using the non-liposomal FuGENE transfection reagent (Roche) as recommended by the manufacturer. Culture supernatants were then assayed for p24 antigen contents 72 hrs post-transfection using an in-house p24 antigen ELISA [28]. Similar results were also obtained with transfected HeLa-tat cells. Virus stocks were then prepared from cleared and filtered culture supernatants (pre-cleared by centrifugation at 1,200 rpm for 7 min and filtered through a 0.45-μm-pore-size membrane) treated with DNase I (Roche) at 20 μg/ml final concentration at 37°C for 1 h. Aliquots in 300-μl fractions of the virus stocks were saved at -80°C until needed. (B) H9 cells (2 × 10^5 ^cells) were infected with the X4 NL4-3 strain of mutant or wild type HIV-1 stocks using 200 ng of p24 antigen per well in 24-well plates. Three hours after infection, unbound viruses were removed by centrifugation, washed and resuspended in 1 ml complete RPMI medium per well. The infections were performed in triplicates and supernatants were collected at days 1, 4, 8, 12 and 16 post-infection and tested for p24 antigen contents by p24-ELISA. NI, non-infected control. (C) For virus binding and internalization assay, monolayered TZM-bl cells were seeded one day before infection and following day, medium was removed and cells were inoculated with equal amounts (400 ng of p24 antigen) of mutant or wild type NL4-3 virus stocks (treated with DNase I) with 20 μg/ml DEAE-dextran (in a total volume of 300 μl to 60,000 cells per well in 12-well plates). After adsorption period of 2 hrs, input viruses were removed and cells were treated with trypsin (+TRYP) or not (-TRYP) and the amount of cell associated p24 was measured using the p24-ELISA. (D) TZM-bl cells were also infected as described above with the amount virus indicated and after adsorption period of 2 hrs, input viruses were removed and cells were fed with 1 ml of complete DMEM with 5 μM indinavir and cultured for 24 hrs. Equal amounts of total RNA isolated from E98A infected TZM-bl cells were subjected to nested RT-PCR using specific primers that amplified a 593 bp fragment of the p17 viral RNA. The outer primer pair 5'-GCA GTG GCG CCC GAA CAG and 5'-TTCTGA TAA TGC TGA AAA CAT GGG TAT and inner primer pair 5'-CTC TCG ACG CAG GAC TC and 5'-ACC CAT GCA TTT AAA GTT CTA G was used. As an internal control, the human β-globin RNA was amplified using the primers described elsewhere [29].

In this study, we investigated the effects associated with two single amino acid substitution mutations, the E98A and E187G respectively, having quite opposite intra molecular CA contacts with other CA residues. The point mutations were engineered by site-directed mutagenesis and as the identity of each mutant was confirmed by sequencing, we assayed the viral protein expression using HeLa-tat and 293T cells [see Additional file 1 for details on Materials & Methods]. We found that the Western blot banding pattern of both mutants were identical to that of wild-type pNL4-3 transfected cells (Fig. 1C). Thus, the mutations did not appear to influence the intra-cellular processing of Gag precursor. We determined by ELISA that cells transfected with the E98A mutant released approximately 15% higher p24 than cells transfected with the control vector (Fig. 2A), indicating that the mutations had no substantial effect on particle release. To determine the viral protein contents of both mutant virions, viral supernatants were concentrated and separated on SDS-PAGE (Fig. 1E and 1F). The samples were then analyzed by immunoblotting with anti-glycoprotein (Fig. 1E) and a pool of HIV positive human sera (Fig. 1F). We observed that virion release was un-affected in both E98A and E187G mutants, as judged by the presence of the intermediate and fully processed Gag proteins [1,2].

**Figure 3:**
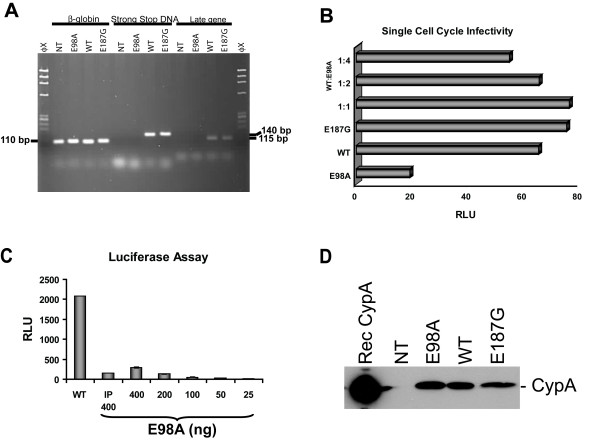
Viral infectivity assay. (A) Detection of proviral DNA. H9 cells were infected as above and total cellular DNA was prepared 16 days post-infection using Qiagen's DNA isolation kit and analyzed by PCR using a set of primers specific for negative strand strong-stop DNA and a conserved region of the *gag*, described previously [30, 31]. Early gene products were amplified using the forward primer Ra 5'-TCT CTG GTT AGA CCA GAT CTG-3' (459–479) and the reverse primer U5a 5'-GTC TGA GGG ATC TCT AGT TAC-3' (584–604). Late gene products representing a conserved region of the HIV-1 *gag *was amplified with the forward primer SK-38 5'-ATC CAC CTA TCC CAG TAG GAG AAA T-3' (1090–1117) and the reverse primer SK-39 5'-TTT GGT CCT TGT CTT ATG TCC AGA ATG C-3' (1177–1204) that amplified a 115-bp fragment. We also examined the viral cDNA production at 16 hrs post-infection and been able to detect in all cells infected with mutant and wild-type virions (data not shown). To normalize for the quantity of total cellular DNA present in each sample, human β-globin DNA was amplified [29]. (B) Single cell cycle infectivity of mutant and wild-type virus particles on TZM-bl reporter cell lines. Cells (2 × 10^4^) were infected as described above with equal amounts (25 ng p24 antigen) of mutant and wild-type virus or chimeric virus stock prepared by co-transfection of mutant and wild-type pNL4-3 at a ratio of 1:1, 2:1, and 4:1. Infected cells were then cultured in the presence of 5 μM indinavir. Twenty-four hours post-infection, cells were harvested in 200 μl Glo lysis buffer (Promega) and assayed for luciferase activity with the luciferase assay kit obtained from Promega. RLU, relative light unit. (C) TZM-bl cells (8 × 10^4^) were infected as described above with 400 ng of wild-type NL4-3 virus or with E98A virus that was first immunoprecipitated with anti-Tat monoclonal antibody (indicated with IP 400). Cells were also infected with E98A virus stock that had been two-fold serially diluted. After 48 hrs, culture supernatants were removed and cells were assayed for luciferase activity. (D) Detection of virion associated cyclophilin A (CypA) by Western blot analysis. Cell free culture supernatants from 293T cells transfected with mutant and wild-type pNL4-3 were equilibrated for p24 antigen concentration and equal amounts of virus was precipitated with Viraffinity (CPG Inc) as recommended by the manufacturer. Culture supernatants were mixed (4:1) with Viraffinity and the mixture was incubated at room temperature for 5 min and centrifuged at 1000 × g for 10 min. The viral pellets were washed and dissolved in 1× RIPA buffer [50 mM Tris/HCl (pH 7.4), 150 mM NaCl, 1% Triton X-100, 1% sodium deoxycholate and 0.1% SDS, supplemented with a complete protease inhibitor cocktail from Roche]. The viral proteins were finally separated by SDS-PAGE, transferred onto a nitrocellulose membrane and probed with rabbit anti-CypA antibody (Calbiochem, 1:2,000) and as secondary antibody horseradish peroxidase-conjugated anti-rabbit IgG. Rec CypA, recombinant CypA; NT, non-transfected control.

However, in contrast to the E187G and wild-type, we found that the E98A virions were non-infectious in permissive CD4 positive H9 cells (Fig. 2B), despite being competent for particle assembly, normal processing of Gag and incorporating viral envelope glycoproteins. Similar results were also seen with infected MT4 cells [see Additional file 2]. The fact that WB analysis of the E98A mutant did not show any defect in proteolytic processing of Gag indicates that the mutation may affect the later stage of virus replication, possibly post-processing. Furthermore, the level of HIV-1 glycoprotein incorporated into the budding virus particle was similar to the wild-type control suggesting that the mutation had no effect at the entry stage of the virus replication cycle. To elaborate this notion, the ability of mutant E98A virus binding and internalization was also determined on CD4^+ ^TZM-bl cells [20], essentially as described elsewhere [21]. Briefly, cells were pre-incubated at 4°C for 1 h and exposed to equal amounts of DNaseI treated E98A or wild-type virus. Following binding at 4°C or internalization at 37°C, cells were treated or not with trypsin and the amount of cell-associated p24 was measured. We observed that mutant E98A virions could bind and internalize into the target cells, indicating that there is no defect at this level of the virus replication cycle (Fig. 2C). Similar results were also seen when the intra-cellular level of viral RNAs were measured using nested RT-PCR (Fig. 2D). In this experiment, TZM-bl cells were seeded and infected as above with two-fold virus dilutions and following internalization, cells were trypsinized, washed and total RNAs were extracted. Equal amounts of RNA were then subjected to nested RT-PCR using specific primers that amplified a 593 bp fragment of the p17 viral RNA. Consequently, in order to determine the exact step at which the viral replication cycle is affected, we used a PCR based system and analyzed the early and late gene replication steps of proviral DNA synthesis *in vivo *in infected cells (Fig. 3A). Infection of H9 cells was performed by addition of cell-free DNaseI-treated virus produced 3 days after transfection of 293T cells. Viral DNA production by E187G mutant virion was at a level similar to that for wild-type pNL4-3. In contrast, we found that viral DNA synthesis in cells infected with E98A virus was completely absent, suggesting that the E98A mutation interferes with an early stage in the viral replication cycle.

Surprisingly, although proviral DNAs in H9 cells infected with E98A virus were not detected, a low level of Tat-induced luciferase activity was detected in a single-cell-cycle infectivity assay with TZM-bl cells (Fig. 3B). Given the fact that Tat is critical for the HIV-1 gene expression and reverse transcription [22,23], we investigated whether a soluble Tat protein released in to the culture supernatant was involved in this assay. To address this issue, possible soluble Tat proteins in the supernatant of transfected HeLa-tat cells was immunoprecipitated using monoclonal antibody against Tat and then tested for the infectivity (Fig. 3C). However, we were unable to inhibit the subtle amount of Tat-induced luciferase activity seen in these cells and subsequently explain this activity. A possible reason may be that Tat is packaged into HIV-1 particles through binding to TAR element [24,25], although the presence of Tat in virion has never been reported satisfactorily. Consistent with a previous report [26], we were also unable to detect Tat proteins in Viraffinity concentrated viral lysate using WB analysis with Tat-specific monoclonal antibody.

Since the E98A mutation is located C-terminal to the CypA-binding site and CypA has been suggested to disrupt CA-CA interactions following cell entry of the virus, we tested whether the reason for the diminished viral replication may be due to the lack of CypA incorporation in to the budding particle. However, analysis of virion-associated proteins revealed similar levels of CypA incorporation as in the control virus (Fig. 3D).

**Figure 4:**
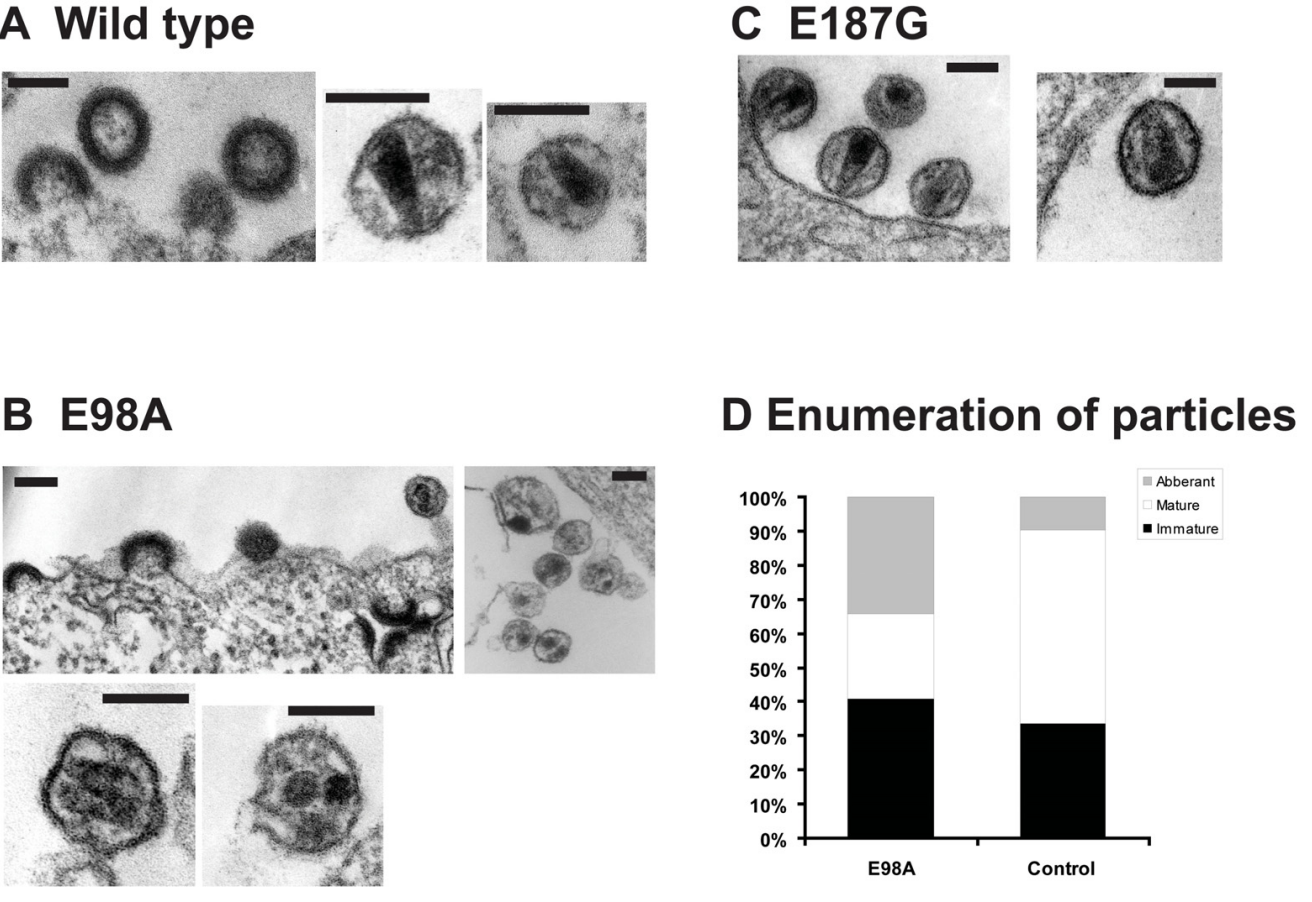
Transmission electron microscopy analysis of mutant and wild-type virions as described previously [4]. (A) With the control virus, a dense core material was shown inside the envelope of immature virus (left panel) and mature virus with dense conical core structure (right panel). (B) Many particles produced by cells transfected with the E98A mutant had either virions with an immature structure or abnormal core morphology (left panel) and a very few detectable cones. Under higher magnification, the E98A virions were observed to be a heterogeneous population of particles (right panel) with varying size and conical core structures, where a number of virions with an electron-lucent centre and aberrant cores were detected (lower panel). (C) E187G virions with a characteristic dense conical core material. Bars, 100 nm. (D) Numerical (%) analysis of 372 wild type NL4-3 and 798 E98A virus particles with respective morphology.

We then examined the morphological structures of these virions and correlated the results to their relative infectivity (Fig. 4). EM images of the three types of particles (NL4-3, E98A, and E187G) were categorized by the presence of three different core structures: aberrant, immature, and mature dense conical structure. Detail morphological analysis was also performed in order to depict different categories of virus morphology [see Additional file 3]. Although small percentage of virus with aberrant core was present, the majority of EM images of NL4-3 and E187G showed a mixture of both mature particles of normal morphology and immature particles (Fig. 4D). In contrast, images of E98A showed mostly aberrant and immature particles (Fig. 4B and 4D). The increased percentage (Fig. 4D) of distorted, aberrant and immature-like E98A virus particles as compared to the wild-type control may thus suggest that the E98 is important for proper protein conformation that is necessary for intermolecular CA-CA interactions. Based on the analysis of inter-atomic contacts [19], we found that the E98 residue is not involved in any inter-atomic contact with other CA residues. Therefore, it is possible to speculate that the E98A mutation may rather be involved in inter-molecular CA-CA interaction or with other possible cellular factors involved in this process.

## List of abbreviations used

HIV, human immunodeficiency virus; CA, capsid; CypA, cyclophilin A;

## Competing interests

The author(s) declare that they have no competing interests.

## Authors' contributions

SA performed most of the experimental work and also wrote the manuscript. SH carried out the electron microscopy analysis. AH assisted SH in electron micrograph analysis and also participated in preparing the illustrations in Figure 1. AV is the principal investigator, conceived of the study, supervised SA and wrote the manuscript together with SA. All authors read and approved the manuscript.

## Supplementary Material

Additional File 1 Materials and Methods. The data provided herein describes in detail the materials and methods used in the study. Click here for file

Additional File 2 Infectivity of mutant and wild-type NL4-3 viruses in MT4 cells. The data provided here describes an additional infectivity assay with mutant and wild-type NL4-3 viruses in MT4 cells. Click here for file

Additional File 3 Detailed electron microscopy analysis of E98A and wild-type NL4-3 virions. The data represents detailed numerical analysis of 798 mutant E98A and 373 wild-type HIV-1 particle morphology. Click here for file
