# Supplementary material for: Mutation in the loop C-terminal to the cyclophilin A binding site of HIV-1 capsid protein disrupts proper virus assembly and infectivity
Source: Retrovirology. 2007 Mar 19;4:19. doi: 10.1186/1742-4690-4-19 (PMC1832212; doi:10.1186/1742-4690-4-19)
Supplement: Additional File 1 — Materials and Methods. The data provided herein describes in detail the materials and methods used in the study. [file 1742-4690-4-19-S1.doc]

**Mutation in the loop C-terminal to the cyclophilin A binding site of HIV-1 capsid protein disrupts proper virus assembly and infectivity**

Samir Abdurahman^1^, Stefan Höglund^2^, Anders Höglund^2^ and Anders Vahlne^1§^

^1^Division of Clinical Microbiology, Karolinska Institutet, Karolinska University Hospial, Stockholm, Sweden. ^2^Department of Biochemistry, Biomedical Center, Uppsala University, Uppsala, Sweden.

**Additional file 1**

**Material and Methods**

**Cells and Reagents**

HeLa-tat, 293T and TZM-bl cells were maintained in Dulbecco's modified Eagle's medium (DMEM; Gibco-Invitrogen) supplemented with 10% fetal bovine serum (FBS) and antibiotics and passaged upon confluence. H9 CD4-positive T-lymphocyte cell lines were grown in RPMI 1640 medium with 10% FBS and antibiotics. DEAE-dextran was purchased from Sigma, recombinant cyclophilin A (CypA) from Boehringer Mannheim GmbH (catalogue no. 1532 219), ABTS from Sigma (catalog no. A9941), recombinant HIV-1 p24 protein from Protein Science inc.(catalog no. 2004), peroxidise-conjugated streptavidine from Jackson Laboratory (catalog no. 016-030-084), rabbit polyclonal antibodies against calnexin from Santa Cruz Biotechnology (catalogue no. sc-11397) and against CypA was from Calbiochem (catalogue no. 239778). The following reagents were obtained through the AIDS Research and Reference Reagent Program, Division of AIDS, NIAID, NIH: HIV-1 Tat monoclonal (8D1.8, catalogue no. 4672) from Dr. Jonathan Karn, the protease inhibitor indinavir sulfate (catalogue no. 8145) and TZM-bl cells (catalogue no. 8129) contributed by Dr. John C Kappes [1].

**Construction of plasmid DNA**

The polymerase chain reaction was utilized to develop all plasmids in the study. QuickChange II XL site-directed mutagenesis kit (Stratagene) was used to mutate the CA sequences using the HIV-1 molecular clone pNL4-3. All plasmid DNAs were propagated in *Escherichia coli* (*E. coli*) XL10-Gold and purified by using a Maxiprep Purification kit (Qiagen) as recommended by the manufacturer. The identity of each mutation was confirmed by sequencing and the resulting plasmids were digested with *Sph*I and *Apa*I. The 563 bp *Sph*I/*Apa*I DNA fragments of the mutated CA sequences were isolated, purified and cloned directionally into the pNL4-3 vector, digested with the same restriction enzymes. The resulting plasmids were propagated in Hb101 competent *E. coli*, purified using Maxiprep purification kit and verified by sequencing.

**Transfection and production of virus stocks**

Transfections of Hela-tat and 293T cells were carried out using the non-liposomal FuGENE transfection reagent (Roche Molecular Biochemicals) as recommended by the manufacturer. Briefly, cells were seeded at 60% confluence one day before and transfected with 2 µg of the infectious molecular clone pNL4-3 and thus mutant derivative (80,000 cells per well in 6-well plates). Cells were harvested 48 to 72 hrs post-transfection in 1× RIPA buffer [50 mM Tris/HCl (pH 7.4), 150 mM NaCl, 1% Triton X-100, 1% sodium deoxycholate and 0.1% SDS, supplemented with a complete protease inhibitor cocktail from Roche] and mixed with SDS sample buffer (0.08 M Tris [pH 6.8], 2.0% SDS, 10% glycerol, 0.1 M dithiothreitol, 0.2% bromophenol blue) before boiling for 5 minutes.

For the production of wild type and mutant viruses, HeLa-tat or 293T cells were transfected as described above. Two to three days after transfection, virion-containing culture supernatants were harvested, pre-cleared by centrifugation at 1,200 rpm for 7 min and filtered through a 0.45-µm-pore-size membrane. Cleared culture supernatants were then treated or not with DNase I (Roche Applied Science) at 20 µg/ml final concentration at 37°C for 1 h and aliquots in 300- μl fractions were saved at -80°C until needed. The p24 concentrations of the virus stocks were quantified by HIV-1 p24 antigen ELISA as described below [2].

**Enzyme-linked immunosorbent assay**

The p24 antigen in transfected or infected culture supernatant or TZM-bl cell lysates were measured using an in-house enzyme-linked immunosorbent assay (ELISA) essentially as described elsewhere [2]. Briefly, 96-well micro well plates (MWP) (Maxisorb, Nunc) were coated with the F919-04-05 rabbit anti-p24 antibody in PBS containing 1 mM phosphate buffer (pH 7.2). Two hundred μl of 3% BSA in PBS was added to the individual wells and blocked for 30 min at 37°C. Subsequently, the wells were incubated with the samples for 1 h at 37°C with a slow agitation or at 4°C overnight. The MWPs were then washed three times with PBS containing 0.05% Tween-20, and incubated with 100 μl of biotinylated rabbit anti-p24 antibody (F919-04-05) in 1% BSA in PBS for 1 h at 37°C. They were then washed three times with PBS containing 0.05% Tween-20, and incubated with peroxidise-conjugated streptavidin (Jacksons Laboratories) for 30 min at 37°C. Finally, the MWPs were washed three times and detected with the enzyme chromogen substrate ABTS (Sigma) in which one tablet was dissolved in 13.3 ml sodium acetate buffer pH4.7. The peroxidise activity was determined by measuring the optical density at 405 nm using a Labsystem multiscan MS spectrophotometer. The concentrations of p24 were calculated from a standard curve derived from a recombinant p24 (Protein Science inc.).

**Virus precipitation**

Virion-associated viral proteins were prepared from cell culture supernatants (normalized for p24 contents) by removal of cellular debris by centrifugation at 1,200 rpm for 7 min and filtering through a 0.45-µm-pore-size membrane. Virus-particle-containing supernatants were then concentrated by centrifugation using Viraffinity (CPC Inc.) as recommended by the manufacturer. Briefly, clarified culture supernatants were mixed with Viraffinity (4:1) and the mixture was incubated at room temperature for 5 min. They were then centrifuged at 1000 × g for 10 min and viral pellets washed 3 times in a buffer containing 60 mM HEPES, 150 mM NaCl, pH 6.5. Finally, the viral pellets were dissolved in 1× RIPA buffer and mixed with SDS sample buffer before being boiled for 5 min.

### Immunoprecipitation

In order to produce virus supernatants free of possible soluble HIV-1 Tat, HeLa-tat culture supernatants (corresponding 400 ng of p24) were clarified of cell debris, adjusted to 500 μl with DMEM and incubated with 20 μl of monoclonal antibody against Tat at room temperature for 2 hrs. Thereafter, 50 μl protein A/G-agarose (Santa Cruz Biotechnology) was added and the suspension was incubated further for 1 h at room temperature. Virus containing culture supernatant free of soluble Tat was collected by centrifugation at 2000 r.p.m. for 5 min and used to infect TZM-bl cells as described above.

**HIV-1 protein analysis**

Denatured whole cell lysates or viral lysates (normalized for p24 contents in the culture supernatant) were separated by SDS-polyacrylamide gel electrophoresis (PAGE). Proteins were then electrophoretically transferred to a nitrocellulose membrane and probed with either HIV-1-positive patient serum (1:200) or polyclonal serum against p24 (1:1,000), calnexin (1:1,000), or Cyclophilin A (1:2,000 [Calbiochem]). Proteins were detected using an appropriate horseradish peroxidase (HRP)-conjugated secondary antibodies raised against mouse (DAKO, 1:4000), human (Pierce, 1:20,000), or rabbit (Sigma, 1:4,000) IgG.

### Infectivity assay

Virus stocks were prepared as described above. H9 cells were infected with the X4 NL4-3 strain of mutant or wild type HIV-1 using 200 ng of p24 antigen. Three hours after infection, unbound viruses were removed by centrifuging the cells at 1,200 rpm for 7. Cells were washed and resuspended in complete RPMI medium and incubated further at 37°C in a 5% CO_2_ incubator. The infections were performed in triplicates, using approximately 2 × 10^5^ cells per well in a final volume of 1 ml. Supernatants were collected at days 1, 4, 8, 12 and 16 post-infection and tested for p24 contents by p24-ELISA. At the end of the assay, cells were also pelleted, washed twice with PBS and analysed by PCR using specific primers that detects the early and late viral gene replication.

### Detection of proviral DNAs

Total DNA was isolated from infected H9 cells sixteen days post-infection. The DNAs were isolated using Qiangen's DNA purification kit as recommended by the manufacturer. An equal amount of DNA from the cells was then subjected to PCR amplification using a set of primers corresponding to the early and late gene replication steps of proviral DNA synthesis. Early gene products were amplified using the forward primer Ra 5'-TCT CTG GTT AGA CCA GAT CTG-3' (459-479) and the reverse primer U5a 5'-GTC TGA GGG ATC TCT AGT TAC-3' (584-604) described previously [3]. Late gene products representing a conserved region of the HIV-1 *gag* was amplified with the forward primer SK-38 5'-ATC CAC CTA TCC CAG TAG GAG AAA T-3' (1090-1117) and the reverse primer SK-39 5'-TTT GGT CCT TGT CTT ATG TCC AGA ATG C-3' (1177-1204) [4] that amplified a 115-bp fragment. To quantify the total cellular DNA present in each sample, human β-globin DNA was amplified using a set of primers PC03 and PC04, which amplified the corresponding sequences of a 110-bp fragment of the first exon of the human β-globin gene [5].

One microgram of each DNA isolated was used as the template for PCR with PCR mastermix (Promega) and were subjected to 29 cycles with denaturation for 1 min at 94°C, primer annealing for 1 min at 55°C, extension for 1 min at 72°C, and 10 min extension step at 72°C. Twenty µl of the amplified PCR products was subjected to agarose gel electrophoresis, and the gel was stained with ethidium bromide.

**Virus binding and internalization assay**

TZM-bl cell line is a HeLa cell clone engineered to stably express CD4, CXCR4 and CCR5, and contain an integrated copy of HIV-1 long terminal repeat (LTR) linked to a luciferase and β-galactosidase gene [1]. Expression of the indicator luciferase gene is under the control of Tat protein that is activated by Tat protein synthesized from the infecting virus.

For virus binding and internalization assay, TZM-bl cells (6×10^4^ cells per well in 6-well plates) were infected essentially as described elsewhere [6]. Briefly, cells were seeded one day before and infected with 400 ng of mutant E98A and wild type NL4-3 virus (treated with DNase I) in the presence of 20 μg/ml DEAE-dextran. To measure virus attachment, cells were incubated at 4°C for 1 h and infected with the mutant E98A and wild type virus. For internalization study, same conditions as above were used except that upon removal of unbound virus, cells were transfered to 37°C and further incubated for 2 hrs. At the end of each procedure, input viruses were removed and cells were treated or not with trypsin, washed and harvested in 0.5× RIPA buffer. The amount of cell associated p24 was measured using an inhouse p24-ELISA.

Cells were also infected with serially, two-fold diluted E98A viruses (400, 200, 100, and 50 ng p24 antigen) for 2 hrs at 37°C. At the end of the assay, cells were trypsinized, washed with PBS and total RNA was extracted using Qiagens’s RNA isolation kit. Equal amount of RNA were then subjected to RT-PCR.

**Nested RT-PCR**

Equal amounts of total RNA isolated from E98A infected TZM-bl cells were subjected to nested RT-PCR using specific primers that amplified a 593 bp fragment of the p17 viral RNA. The outer primer pair 5’-GCA GTG GCG CCC GAA CAG and 5’-TTCTGA TAA TGC TGA AAA CAT GGG TAT and inner primer pair 5’-CTC TCG ACG CAG GAC TC and 5’-ACC CAT GCA TTT AAA GTT CTA G was used. The outer primer pair was used to detect the viral RNA by first synthesizing complementary DNA (cDNA) in the presence of retroviral reverse transcriptase. Following this reaction, the cDNA products were subjected to a new cycle of PCR to detect the 593 bp fragment of the viral RNA.

**Single cell cycle infectivity**

For relative viral infectivity assay, TZM-bl cells were seeded one day before infection. Following day, medium was removed and target cells were inoculated by adding mutant and wild type NL4-3 virus or chimeric virus stocks prepared by co-transfection of mutant and wild-type pNL4-3 at a ratio of 1:1, 2:1, and 4:1. The cells were infected with a virus stocks corresponding to 25 ng of p24 antigen with 20 μg/ml DEAE-dextran (in a total volume of 300 μl to 20,000 cells per well in 12-well plates).

Since we observed a subtle amount of Tat activity with the E98A virions in this assay, we also tested the infectivity of E98A virions that were first immunoprecipitated with anti-Tat monoclonal antibody as described above. In this assay, the cells (80 000 per 6-well culture plate) were infected with 400 ng of wild-type NL4-3 virus or with E98A virus that was immunoprecipitated. Cells were also infected with E98A virus that was serially two-fold diluted (400, 200, 100, 50, and 25 ng).

After adsorption period of 2 hrs, input viruses were removed and cells were washed and fed with a complete DMEM containing 5 μM indinavir and cultured for 24 to 48 hrs. At the end of the assay, culture supernatants were removed and cells were assayed for luciferase activity with the luciferase assay kit obtained from Promega as recommended by the manufacturer. Briefly, culture supernatants were removed and 200 to 400 μl Glo Lysis Buffer (Promega) was added to each well, and incubated for 5 min at room temperature to allow complete cell lysis. One hundered μl of the cell lysate was transfered to a white 96-well micro well plate (Costar) and a volume of Bright-Glo™ Reagent (Promega) equal to that of the cell lysate was added before measuring the luminescence using the Luminoskan Ascent luminometer (ThermoLabsystem).

**Transmission electron microscopy (TEM) analysis of HIV-1**

Transfected HeLa-tat cells were fixed freshly upon embedding in epon, essentially as described before [7]. Importantly, sections were made approximately 60 nm thick to allow accommodation of the volume of the core structure parallel to the section plane. Duplicate sample preparations were done as a control and minimal beam dose technique was employed throughout. Enumeration based on morphology was done with series of E98A electron micrographs to depict different categories of virus morphology, specifically focusing on the packing of the virus core structure.

**References**

1. Wei X, Decker JM, Liu H, Zhang Z, Arani RB, Kilby JM, Saag MS, Wu X, Shaw GM, Kappes JC: **Emergence of resistant human immunodeficiency virus type 1 in patients receiving fusion inhibitor (T-20) monotherapy.** *Antimicrob Agents Chemother* 2002, **46:**1896-1905.

2. Horal P, Svennerholm B, Jeansson S, Rymo L, Hall WW, Vahlne A: **Continuous epitopes of the human immunodeficiency virus type 1 (HIV-1) transmembrane glycoprotein and reactivity of human sera to synthetic peptides representing various HIV-1 isolates.** *J Virol* 1991, **65:**2718-2723.

3. Jacque JM, Triques K, Stevenson M: **Modulation of HIV-1 replication by RNA interference.** *Nature* 2002, **418:**435-438.

4. Ou CY, Kwok S, Mitchell SW, Mack DH, Sninsky JJ, Krebs JW, Feorino P, Warfield D, Schochetman G: **DNA amplification for direct detection of HIV-1 in DNA of peripheral blood mononuclear cells.** *Science* 1988, **239:**295-297.

5. Saiki RK, Scharf S, Faloona F, Mullis KB, Horn GT, Erlich HA, Arnheim N: **Enzymatic amplification of beta-globin genomic sequences and restriction site analysis for diagnosis of sickle cell anemia.** *Science* 1985, **230:**1350-1354.

6. Guyader M, Kiyokawa E, Abrami L, Turelli P, Trono D: **Role for Human Immunodeficiency Virus Type 1 Membrane Cholesterol in Viral Internalization.** *J Virol* 2002, **76:**10356-10364.

7. Hoglund S, Su J, Reneby SS, Vegvari A, Hjerten S, Sintorn IM, Foster H, Wu YP, Nystrom I, Vahlne A: **Tripeptide interference with human immunodeficiency virus type 1 morphogenesis.** *Antimicrob Agents Chemother* 2002, **46:**3597-3605.
