# Supplementary material for: Mutation in the loop C-terminal to the cyclophilin A binding site of HIV-1 capsid protein disrupts proper virus assembly and infectivity
Source: Retrovirology. 2007 Mar 19;4:19. doi: 10.1186/1742-4690-4-19 (PMC1832212; doi:10.1186/1742-4690-4-19)

**Mutation in the loop C-terminal to the cyclophilin A binding site of HIV-1 capsid protein disrupts proper virus assembly and infectivity**

Samir Abdurahman^1^, Stefan Höglund^2^, Anders Höglund^2^ and Anders Vahlne^1§^

^1^Division of Clinical Microbiology, Karolinska Institutet, Karolinska University Hospial, Stockholm, Sweden. ^2^Department of Biochemistry, Biomedical Center, Uppsala University, Uppsala, Sweden.

**Additional file 2**

Western blot analysis of infected cells. MT4 cells (4×10^5^) were infected with the X4 NL4-3 strain of mutant and wild type HIV-1 using 200 ng of p24 antigen per well in 24-well plates. Cells were also infected with chimeric viruses produced by transfection of mutant and wild-type pNL4-3 as indicated. Three hours after infection, unbound viruses were removed by centrifuging the cells at 1,200 rpm for 7 min. Cells were then washed, resuspended in complete RPMI medium and incubated further at 37°C in a 5% CO_2_ incubator. At day four, culture supernatants were removed and cells were lysed in 1× RIPA buffer [50 mM Tris/HCl (pH 7.4), 150 mM NaCl, 1% Triton X-100, 1% sodium deoxycholate and 0.1% SDS, supplemented with a complete protease inhibitor cocktail from Roche]. Denatured cell lysates were then separated by SDS-PAGE in 10-20% gels and transferred to a nitrocellulose membrane. The viral proteins in the membrane were initially detected with HIV+ patient sera (A) and was then reprobed with rabbit anti-calnexin and anti-p24 antibodies (B). NI, non-infected control. The positions of specific viral proteins and calnexin are indicated in the middle. (C) The viral p24 antigen contents in the infected culture supernatants were also assayed using p24-ELISA.


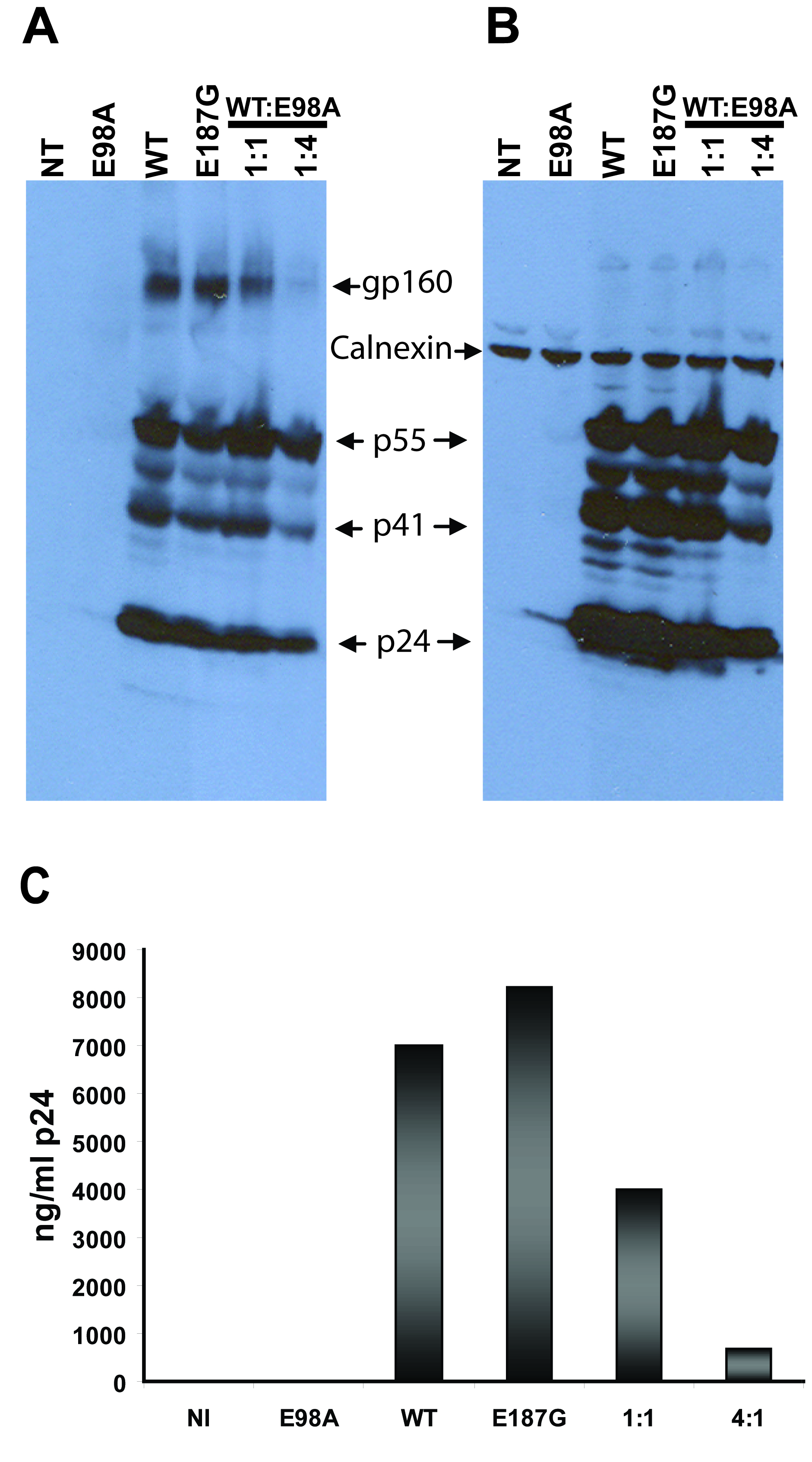

Supplement: Additional File 2 — Infectivity of mutant and wild-type NL4-3 viruses in MT4 cells. The data provided here describes an additional infectivity assay with mutant and wild-type NL4-3 viruses in MT4 cells. [file 1742-4690-4-19-S2.doc]
