# Supplementary material for: Mutation in the loop C-terminal to the cyclophilin A binding site of HIV-1 capsid protein disrupts proper virus assembly and infectivity
Source: Retrovirology. 2007 Mar 19;4:19. doi: 10.1186/1742-4690-4-19 (PMC1832212; doi:10.1186/1742-4690-4-19)
Supplement: Additional File 3 — Detailed electron microscopy analysis of E98A and wild-type NL4-3 virions. The data represents detailed numerical analysis of 798 mutant E98A and 373 wild-type HIV-1 particle morphology. [file 1742-4690-4-19-S3.doc]

**Mutation in the loop C-terminal to the cyclophilin A binding site of HIV-1 capsid protein disrupts proper virus assembly and infectivity**

Samir Abdurahman^1^, Stefan Höglund^2^, Anders Höglund^2^ and Anders Vahlne^1§^

^1^Division of Clinical Microbiology, Karolinska Institutet, Karolinska University Hospial, Stockholm, Sweden. ^2^Department of Biochemistry, Biomedical Center, Uppsala University, Uppsala, Sweden.

**Additional file 3**

EM analysis of E98A and wild-type virions. We performed detailed analysis on core morphology of 798 mutant E98A and 372 wild-type HIV-1 particles. The results were presented as two different categories of virus morphologies: aberrant and mature plus immature core structures. A numerical analysis of 372 wild type NL4-3 particle images with respective morphology revealed that 336 (90%) showed mature and immature phenotype; whereas the E98A sample set consisted of 798 particles showed only 542 (66%) mature and immature core structures. However, the number of virus with a typical mature core morphology between the two groups were markedly different, the wild type control contained 48 (13%) particles with mature-like morphology whereas the number of this particles were only 18 (2%) of the E98A particles. Furthermore, 272 (34%) of the E98A particle images showed aberrant core morphology as compared to 36 (9%) in the control virus.
